# Potential role of N6-methyladenosine modification in circular RNA biogenesis and function in the inflammatory responses

**DOI:** 10.3389/fmmed.2025.1607661

**Published:** 2025-06-26

**Authors:** Heedoo Lee, Leo Chen, Yang Jin

**Affiliations:** ^1^ Department of Medicine, Division of Pulmonary and Critical Care Medicine, Boston University, Boston, MA, United States; ^2^ Department of Biology and Chemistry, Changwon National University, Changwon, Republic of Korea

**Keywords:** fungal infection, lung, pneumonia, lung injury, inflammation

## Abstract

N6-methyladenosine (m6A) is the best-studied post-transcriptional RNA modification. It refers to the methylation in the N6 position. M6A exists universally from viruses to mammalian cells and is highly abundant in RNA polymerase II-transcribed, protein-coding transcripts and various non-coding RNAs. M6A RNA modification influences multiple physiological and pathological processes. This RNA methylation plays a role in the pathogenesis of many human diseases, including but not limited to hematopoietic, central nervous, and reproductive systems. One of the m6A-modified non-coding RNAs is the circular form of RNA. Circular RNA (circRNA) refers to a single-stranded RNA molecule with a circular structure that exists across a wide range of organisms, including eukaryotes and prokaryotes. Its unique circular structure is formed by the covalent closure between the 3′and 5′ends of the RNA molecule. This closed-loop structure prevents the circRNA from being degraded readily by the exonucleases, resulting in more stability compared to its linear RNA counterparts. CircRNAs have been reported to regulate gene expression, protein interaction, and RNA sponging. They play important roles in many human diseases. M6A modifications of the host gene mRNAs regulate the circRNA biogenesis. Furthermore, m6A modification of circRNA itself adds additional regulation of these complicated processes. This mini-review elaborates on recent advances in m6A modification on circRNA biogenesis and function, focusing on the role of circRNA m6A modification in the development of inflammatory responses.

## Introduction

N6-methyladenosine (m6A) refers to the methylation in the N6-position of adenosine, the most abundant post-transcriptional chemical modification of RNAs. It is estimated that approximately 1,000 nucleotides on average contain one to two m6A residues (Beemon and Keith, 1977; Krug et al., 1976). M6A exists in the RNA of bacteria, viruses, and mammalian cells (Desrosiers et al., 1974; Deng et al., 2015). It is highly prevalent in RNA polymerase II (RNAPII) transcribed, protein-coding transcripts, and non-coding RNAs. M6A RNA modification influences various physiological and pathological processes. This RNA methylation plays a role in the pathogenesis of many human diseases, including but not limited to hematopoietic, central nervous, and reproductive systems. M6A is also well-studied in tumorigenesis (Jiang et al., 2021; Fan et al., 2023; Mu et al., 2024). In this mini-review, we elaborate on recent advances in m6A modification on circRNA biogenesis and function. We also highlight the underlying mechanism of m6A in circRNA formation.

### History of m6A research

In 1955, DB Dunn and JD Smith first reported an m6A base in bacterial DNA (Dunn and Smith, 1955). In 1958, more studies reported the presence of m6A in bacterial and yeast RNAs (Littlefield and Dunn, 1958; Adler et al., 1958). Rapid-growing findings on m6A emerged after the more efficient mRNA isolation techniques. In the 1970s, m6A was identified in mammalian RNAs were discovered (Desrosiers et al., 1974). From the 1990s to the 2010s, the m6A RNA methyltransferase writer complex, m6A reader, and eraser complex were defined, followed by more understanding of the essential roles of m6A in human disease processes (Liao et al., 2021; Bokar et al., 1994; Bokar et al., 1997; Liu et al., 2014; Liu et al., 2015; Ping et al., 2014; Wang et al., 2014b; Wang et al., 2016; Wang P. et al., 2016; Śledź and Jinek, 2016). Another milestone of m6A research is the development of the Global maps of m6A methylation (Sendinc and Shi, 2023). Currently, it is well understood that in addition to mRNAs, abundant m6A methylation is discovered in a variety of non-coding RNAs (ncRNAs) (Brown et al., 2016; Liu N. et al., 2013; Pendleton et al., 2017; Warda et al., 2017; Linder et al., 2015; Meyer et al., 2012). The corresponding enzymes that mediate m6A on ncRNAs are identified, e.g., ZCCHC4, METTL5, METTL16, and METTL4 (Schöller et al., 2018). The enzyme PCIF1 is identified on mRNAs (Sendinc et al., 2019). The ncRNAs regulated by m6A modification include but are not limited to microRNAs (miRNAs), long non-coding RNAs (lncRNAs), circRNAs, small nuclear RNAs (snRNAs), small nucleolar RNAs (snoRNAs), and ribosomal RNAs (rRNAs) (Desrosiers et al., 1974; Alarcón CR. et al., 2015; Liu N. et al., 2017; Yang et al., 2017). In this review, we will focus on the role of m6A in circRNAs.

### Regulation of m6A

**SCHEME 1:**
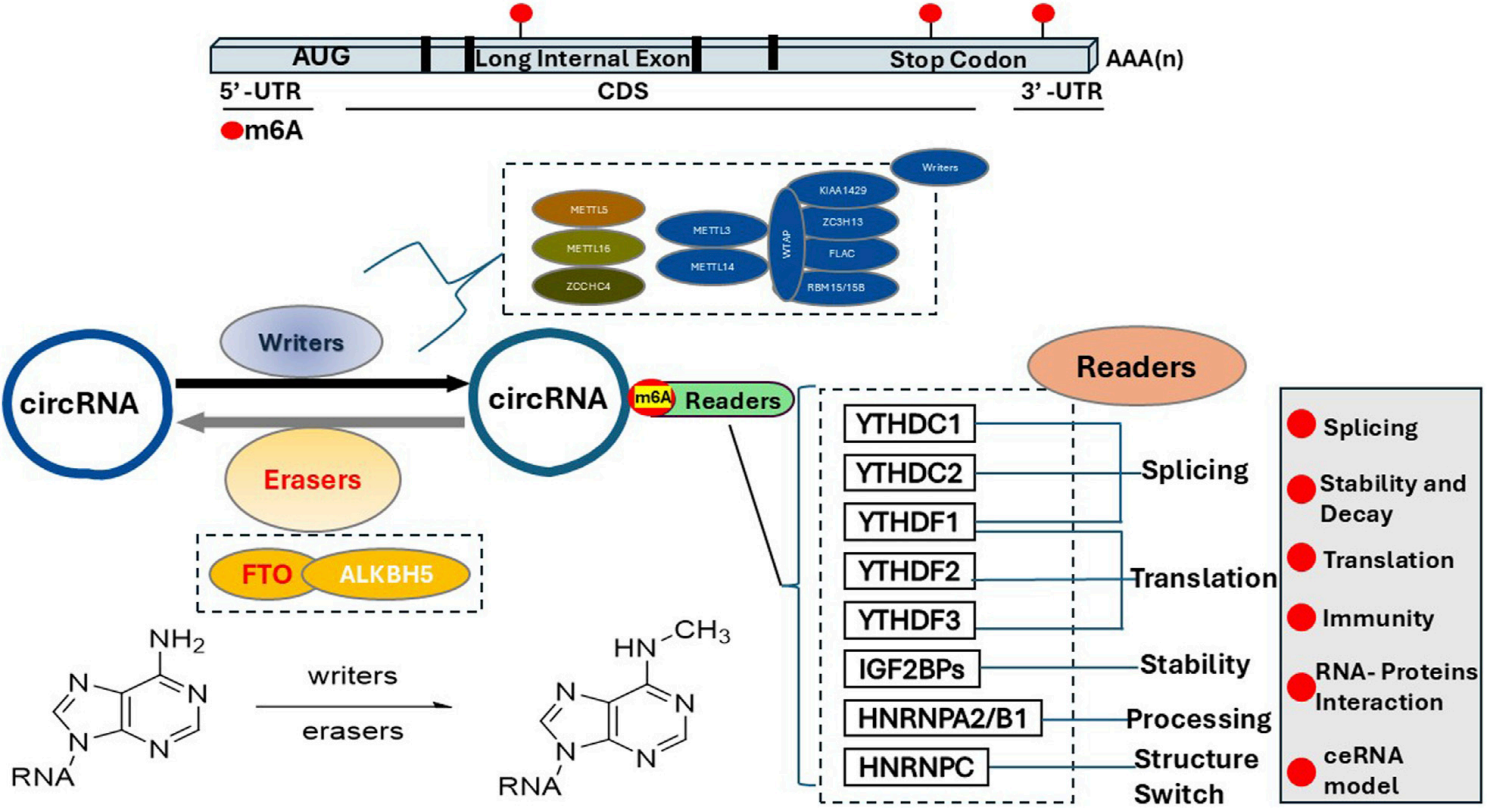
M6A location, formation, recognition and deletion. M6A is enriched in the 3′untranslated region (3′UTR), around stop codons, and within long internal exons, particularly in regions with the consensus sequence RRACH. The primary m6A writer to introduce m6A modification on the circRNAs is the METTL3 complex, with METTL3 being the catalytic subunit and METTL14 acting as the RNA-binding platform. Other proteins involved in the m6A writer complex are shown here, e.g., WTAP, RBM15, and ZC3H13. Currently, the reported m6A eraser includes FTO (fat mass and obesity-associated protein) and ALKBH5, which reverse m6A modifications. M6A readers are proteins that recognize and bind to m6A modifications on RNA, regulating RNA metabolism and gene expression by influencing processes like splicing, stability, translation, and localization. The m6A readers include but not limited to the YTH domain family (YTHDF1-3, YTHDC1-2), IGFBPs (insulin-like growth factor 2 mRNA-binding proteins), MSI2 (Musashi2), PRRC2A (Proline-rich coiled-coil containing protein 2 A), and HNRNPA2B1 (Heterogeneous nuclear ribonucleoprotein A2/B1).

The regulation of m6A is conducted via three major complexes, i.e., m6A writer complex, m6A reader proteins, and m6A eraser enzymes. M6A writer includes METTL3, METTL14, WTAP, VIRMA, RBM15, and ZC3H13 (Wu et al., 2016; Xu et al., 2017; Zhang et al., 2020; Xiang et al., 2017; Visvanathan et al., 2018; Zhou et al., 2021; Gu et al., 2019; Yang X. et al., 2020; Du et al., 2021). The demethylase FTO or ALKBH5 is responsible for removing m6A modification, i.e., m6A erasers (Qu et al., 2022; Yu et al., 2023; Gao et al., 2024; Tsuchiya et al., 2022). M6A reader proteins recognize and interact with m6A on RNA molecules, thereby regulating RNA splicing, stability, translation, and nuclear export, ultimately influencing the fate of the mRNA and gene expression. M6A reader essentially “interprets” the m6A mark on RNA to direct its cellular function. The summary of the m6A writer, eraser, and readers is illustrated in Scheme 1.

### M6A writers

The development of global mRNA m6A methylation mapping suggests that m6A is enriched near the stop codon, 3′untranslated region (UTR), and long internal exon (Dominissini et al., 2012; Meyer et al., 2012). M6A mainly occurs in a consensus sequence motif (RRACH) (where R = A or G, H = A, C, or U) (Bokar et al., 1997; Wei and Moss, 1977). The two most important components of the m6A writer complex are methyltransferase-like 3 (METTL3) and methyltransferase-like 14 (METTL14), forming the methyltransferase complex (MTC) (Xu et al., 2022; Garcias Morales and Reyes, 2021). This evolutionarily conserved MTC complex also includes VIRMA/Virilizer, WTAP, Hakai, ZC3H13, and a newly discovered writer METTL16 (Knuckles et al., 2018; Su et al., 2022). METTL14, combined with METTL3, plays an essential role in substrate recognition (Liu et al., 2014; Wang et al., 2016; Wang P. et al., 2016). The RNA secondary structure and splicing proteins both may contribute to m6A distribution by preventing m6A methylation (Schwartz et al., 2013) or by potentially blocking access of the METTL3 complex to nascent RNAPII transcripts during transcription (Uzonyi et al., 2023; Yang et al., 2022; He et al., 2023). Among all the components of MTC, METTL3 is the primary RNA methyltransferase enzyme that adds a methyl group to the N6 position of adenosine residues on RNA molecules, i.e., the m6A. METTL14 facilitates the binding of the MTC to adjacent RNA polymerase II and transfers the MTC to actively transcribed nascent RNAs (Huang et al., 2019). In addition to METTL3 and METTL14, Wilms Tumor 1-associated protein (WTAP) connects the METTL3-METTL14 heterodimer to the nuclear speckle and promotes catalytic activity (Schwartz et al., 2014; Ping et al., 2014; Wang et al., 2023a). VIRMA guides m6A to occur close to the stop codon in 3′UTR by recruiting the MTC (Yue et al., 2018). ZC3H13 enhances m6A by bridging WTAP to the mRNA-binding factor Nito (Knuckles et al., 2018; Wen et al., 2018). METTL16 catalyzes m6A modification in U6-snRNA (Warda et al., 2017). It methylates long noncoding RNA (lncRNA) and U6 small nuclear RNA (U6 snRNA) (Brown et al., 2016; Fitzsimmons and Batista, 2019). The METTL16-mediated methylation requires the UACAGAGAA sequence (Doxtader et al., 2018; Mendel et al., 2018). Additionally, the methyl-group donor S-adenosylmethionine (SAM) is also regulated by METTL16 (Pendleton et al., 2017).

### M6A erasers

An “m6A eraser” is a protein that removes the m6A modification from RNA molecules. The m6A marks in the eukaryote RNAs are installed and reversed dynamically and constantly. As mentioned above, the MTC complex is responsible for installing the m6A, while the erasers, e.g., FTO or ALKBH5, are responsible for removing the m6A modifications. The m6A eraser functions as a “demethylase” to regulate gene expression by altering the stability and translation of specific RNA transcripts; Two well-studied m6A erasers are the proteins fat mass and obesity-associated protein (FTO) and alkB homologue 5 (ALKBH5). FTO and ALKBH5 belong to the AlkB family of Fe(II) and α-ketoglutarate-dependent dioxygenases. Among the nine AlkB family homologs, the first eight were labeled as ALKBH1-8, and the ninth was known as FTO (Alemu et al., 2016; Liu C. et al., 2013; Fedeles et al., 2015).

Both FTO and ALKBH5 carry conserved double-stranded β-helix (DSBH) domains to regulate their demethylase activity; they remove alkyl adducts from bases through oxidative demethylation (Fedeles et al., 2015). FTO recognizes and interacts with a specific sequence on the RNA molecule containing the m6A modification, allowing it to access the methyl group for removal. FTO removes the m6A modifications on RNA by directly catalyzing the demethylation reaction, essentially deleting the methyl group from the adenine base and converting it back to regular adenosine, thus preventing m6A “reader” proteins from binding and thereby altering the RNA stability, splicing, and translation levels.

ALKBH1-8 (Fu et al., 2010; Ringvoll et al., 2006; Aas et al., 2003), greatly facilitates the development of inhibitors targeting m6A demethylases. While both FTO and ALKBH5 serve as “erasers” of m6A modifications, their secondary structures and substrates are significantly different (Scheme 1).

### M6A readers

An “m6A reader” is a protein that specifically binds with m6A, and recognizes and interacts with methylated RNA molecules. The m6A reader can influence mRNA stability, translation, and nuclear export via “reading” the m6A mark on RNA to trigger downstream functions. Unlike the m6A erasers, many proteins have been reported to be capable of recognizing and reacting to the m6A marks (Shi H. et al., 2019; Alarcón C. R. et al., 2015; Huang et al., 2018; Du et al., 2016; Shi Y. et al., 2019). One big category of proteins is the YTH domain-containing proteins, including but not limited to: YTHDF1, YTHDF2, YTHDF3, YTHDC1, and YTHDC2. Among them, YTHDF1 mediates target gene expression, YTHDF2 promotes targeted mRNA decay, YTHDF3 facilitates mRNA translation and promotes the decay of m6A-modified RNAs, and YTHDC1 regulates RNA splicing and nuclear export protein. YTHDC2 plays an important role in RNA translation and decay. In addition to the YTH domain-containing proteins, HNRNPA2B1, HNRNPC, NKAP, IGF2BP1, IGF2BP2, IGF2BP3, fragile X mental retardation protein (FMRP), eukaryotic initiation factor 3 (eIF3), HuR, CNBP all have been reported to function as m6A readers (Shi H. et al., 2019; Alarcón C. R. et al., 2015; Huang et al., 2018; Du et al., 2016; Shi Y. et al., 2019). They identify and interpret m6A sites on diverse transcripts to regulate the fate of target mRNAs, and subsequently regulate RNA metabolism, tumorigenesis, hematopoiesis, viral replication, immune response, and adipogenesis. Recently, one of the m6A writers, METTL16, was also reported to serve as a reader and participate in catalyzing m6A in A43 of the U6 small nuclear RNA (Warda et al., 2017).

### Function of m6A modifications

The cellular function of m6A has been well illustrated. M6A affects the stability, splicing, and translation of RNA and enhances the degradation of specific transcripts. Consequently, m6A alters the downstream signaling or transcription of the targeted mRNAs and regulates the expression of genes that affect growth, development, and other biological functions. Biologically, m6A has been reported as a tumor suppressor or promoter (Huang et al., 2020). It also plays a role in neuron injury, axonal regeneration, and malformation (Weng et al., 2018; Wang D. et al., 2023; Yang C. et al., 2020). M6A can regulate the cell differentiation of hematopoietic stem cells, stem cell self-renewal, DNA damage response, neurological function, and sex determination. Furthermore, m6A methyltransferases mediate therapy resistance to chemotherapy, targeted therapy, immunotherapy, and radiotherapy.

In addition to the impact on the protein-coding RNAs, emerging influences of m6A on non-coding RNAs have been reported.

### M6A regulation of ncRNA biogenesis and function

Non-coding RNAs (ncRNA) make up the majority of total RNAs in mammals. In humans, it is estimated that over 90% of transcribed RNA is non-coding. Currently, more than 18,000 distinct ncRNAs have been reported. The most abundant ncRNAs are ribosomal RNAs (rRNA) and transfer RNAs (tRNA) (Zhou R. et al., 2020; Kaikkonen et al., 2011; Sharma et al., 2024; Dahariya et al., 2019; Hung et al., 2020; Parashar et al., 2022). Besides rRNAs and tRNAs, based on their sizes, ncRNAs are divided into two major groups: short noncoding RNAs (18–200 nucleotides) and long ncRNAs (lncRNAs) (>200 nucleotides). In addition, a novel class of ncRNAs was recently discovered, the circRNAs (Zhou R. et al., 2020; Feng et al., 2023; Santer et al., 2019). The size of circRNAs ranges from less than 200 to several thousand nucleotides. Examples of short ncRNAs include microRNAs (miRNAs) and small nuclear RNAs (snRNAs). MiRNAs have only 22 to 25 nucleotides (Ranganathan and Sivasankar, 2014; M et al., 2005; Kim and Nam, 2006; Saini et al., 2007). Simply to the impacts on protein-coding mRNAs, m6A modification on non-coding RNA (ncRNA) significantly impacts its stability, function, and interactions with proteins, resulting in various biological processes, including but not limited to cell differentiation, development, and pathogenesis of human diseases. In this mini-review, we will focus on the current advances in the role of m6A in the regulation of the newly discovered circRNAs, and the impact of circRNA m6A modification in the development and resolution of inflammatory responses, particularly after bacterial infections.

### The impact of m6A on circRNA biogenesis

**SCHEME 2:**
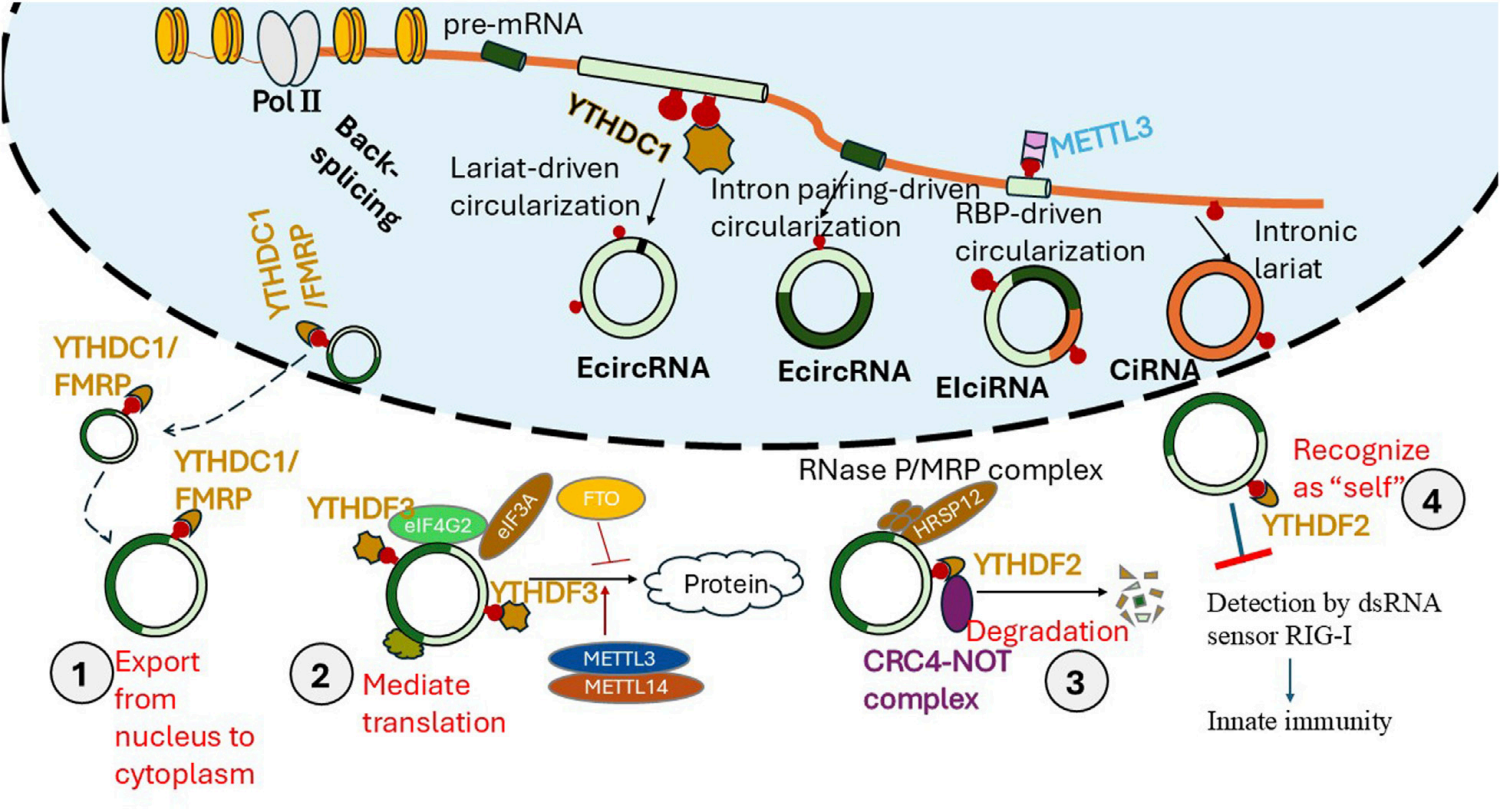
M6A participates in the circRNA biogenesis, export, and function. CircRNA biogenesis primarily occurs through “back-splicing,” where the 5′end of an exon is joined to the 3′end of either the same or an upstream exon, creating a closed loop. This process is mediated by the spliceosome, and facilitated by specific RNA structures and RNA-binding proteins (RBPs). There are several types of “back-splicing”. As illustrated in the schema, Intronic CircRNAs (ciRNAs) refer to the circRNAs that are formed from introns themselves. Exonic circRNAs (EcircRNAs) contain only exons and no introns. Exonic-intronic circRNAs (EIciRNAs) contain both exons and introns. In certain cases, circRNAs can be formed through a lariat-driven mechanism, where exon skipping removes the exons to be back-spliced, and the splice signals of the circRNA exons are juxtaposed in the lariat structure. M6A regulates CircRNA Biogenesis (1), CircRNA Stability and Degradation (2) CircRNA Translation (3), CircRNA Localization, Biological Functions (4). M6a formation of the host RNA facilitated the biogenesis of circRNAs, by regulating the splicing and circularization processes, e.g., m6A sites near the start and stop codons can recruit spliceosomes, leading to back-splicing and circRNA formation. M6A modification affects the stability and degradation of circRNAs. M6A-modified circRNAs also are recognized by specific reader proteins (like YTHDF2) and targeted for degradation by the RNase P/MRP complex. M6A modification regulates circRNA translation, particularly in a cap-independent manner. M6A residues can drive efficient translation initiation from circRNAs, requiring proteins like eIF4G2 and YTHDF3. M6A modification can influence the nuclear-cytoplasmic transport of circRNAs. In the nucleus, m6A can bind to specific reader proteins, like YTHDC1, promoting circRNA export to the cytoplasm. m6A-modified circRNAs participate in various physiological and pathological processes, including immunity, tumor development, and other diseases. For example, m6A-modified circRNAs can participate in tumor immune surveillance. Endogenous circRNAs modified by m6A can be recognized as “self” by m6A reader YTHDF2, subsequently block RIG-1 activation and innate immune responses.

Unlike linear RNAs, circRNA is a single-stranded RNA that forms a covalently closed continuous loop (Yang et al., 2017; Feng et al., 2023; Santer et al., 2019; Pisignano et al., 2023; Kristensen et al., 2019; Zhou WY. et al., 2020). They are often conserved across species in mammals and have tissue/cell specificity (Yang et al., 2017; Feng et al., 2023; Santer et al., 2019; Pisignano et al., 2023; Kristensen et al., 2019; Zhou WY. et al., 2020). CircRNAs are generated via back-splicing from their linear host mRNAs (Scheme 2). CircRNAs do not have 5′ or 3′ ends. Due to the circular structure, their half-life is much longer as circRNAs are more resistant to exonuclease-mediated degradation (Yang et al., 2017; Feng et al., 2023; Santer et al., 2019; Pisignano et al., 2023; Kristensen et al., 2019; Zhou WY. et al., 2020). Emerging evidence has indicated that circRNAs function as gene regulators and can encode functional proteins/peptides. CircRNAs can also serve as potential prognostic markers or therapeutic targets in various human diseases. However, the function of most circRNAs remains unclear, particularly in sepsis-associated lung inflammation.

Accumulating evidence suggests that m6A modification significantly impacts circRNA biogenesis.

As mentioned above, the back-splicing is crucial for the formation of circRNAs. The interaction with specific “m6A reader”, like YTHDC1, m6A promotes the production of certain circRNAs and affects their stability and cytoplasmic export; M6A can either enhance or suppress the generation of specific circRNAs depending on the location of the m6A modification.

### M6A regulates the back-splicing of circRNAs

According to current research, m6A modification on host mRNA can promote the formation of circRNAs by modulating the splicing process and facilitating back-splicing, which is the key mechanism for circRNA generation. The presence of m6A marks enhances the likelihood of a pre-mRNA forming a circular structure.

In 2020, Timoteo et al. (2020) demonstrated that m6A modifications control the circRNA metabolism: m6A can regulate whether a specific exon undergoes linear or back-splicing, and correlate with cap-independent translation of circ-ZNF609 (Di Timoteo et al., 2020). They further showed that the altered expression of m6A writer METTL3 and m6A reader YTHDC1 might contribute to the observed circ-ZNF609 upregulation. Same year 2020, Tang et al. (2020) reported that m6A promotes the biogenesis of circRNA in male germ cells (Tang et al., 2020). For open reading frames (ORFs)-containing circRNAs during murine spermatogenesis, the back splicing occurred mostly at m6A-enriched sites. They found that these m6A sites were usually located around the start and stop codons in linear mRNAs. Tang et al. (2020) deleted ALKBH5 and METTL3, respectively. After deleting ALKBH5 (m6A eraser) in spermatogenic cells, the m6A level was significantly increased compared with wild-type controls, and consistently, the circRNAs abundance is markedly increased in Alkbh5-null spermatogenic cells (Tang et al., 2020). After deleting METTL3 (m6A writer), much fewer circRNAs were identified.

Following studies by Dattilo et al. (2023) further emphasized the importance of m6A reader YTHDC1 in the back splicing and formation of circRNAs. The RNA helicase DDX5 functions as a mediator of the back-splicing reaction and as a co-factor of the m6A regulatory network. DDX5 and the m6A reader YTHDC1 interact and promote the production of a common subset of circRNAs. M6A modification at specific sites, particularly those near the start and stop codons, can recruit YTHDC1 and spliceosomes, which then promotes the precursor transcript towards circularization by driving the back-splicing reaction and leading to circRNA production.

### M6A regulates the cellular function of circRNAs

M6A modification can be identified not only on host mRNA which are precursors of circRNAs, but also on the mature circRNAs themselves. The m6A modification on mature circRNAs exerts essential roles in the transport, metabolism, degradation, and function of the circRNAs.

### M6A regulates the transport of circRNAs

In the nucleus, the m6A can bind YTHDC1 and subsequently promote the export of circRNAs. Upon circRNAs export to the cytoplasm, m6A binds to specific reader proteins to stabilize some mRNAs (Chen RX. et al., 2019). Therefore, the nuclear export of circRNAs also affects its miRNA sponges. M6A modification can also promote the cytoplasmic export of circRNAs, facilitating their localization and function in the cytoplasm. For example, m6A modification of circNSUN2 increases its cytoplasmic export in colorectal cancer (Chen RX. et al., 2019).

### M6A regulates the degradation of circRNAs

Unlike mRNA, circRNAs have a covalently closed loop and do not have a 3′polyadenylated tail, so they are naturally more stable than their homologous linear RNAs (Liu L. et al., 2017; Park et al., 2019). CircRNAs can only be degraded by endoribonucleolytic cleavage. Park et al. (2019) reported that circRNAs containing m6A can be decayed through YTHDF2-HRSP12-RNase P/MRP-mediated endoribonucleolytic cleavage. The abundance of circRNAs containing m6A increased after a component of RNase P/MRP was downregulated (Park et al., 2019). YTHDF2 is a YTH-domain-containing protein that can recognize and destabilize m6A-containing RNAs. HRSP12 (human heat-responsive protein 12)- Eukaryotic RNase-P and RNase MRP are essential ribonucleoprotein complexes that function as endoribonucleases. The m6A-containing circRNAs interacted with YTHDF2 in an HRSP12-dependent manner. HRSP12 is an adapter to bridge YTHDF2 and RNase-P/MRP, inducing rapid degradation of YTHDF2-bound circRNAs (Legnini et al., 2017).

### M6A regulates the translation of circRNA

CircRNA has been frequently considered as non-coding RNA. However, emerging evidence has shown that some circRNAs can be translated into proteins (Yang et al., 2017; Legnini et al., 2017; Pamudurti et al., 2017; Zhang et al., 2018a; Liang et al., 2019; Huang et al., 2021; Zhang et al., 2018b) reported a database, TransCirc (Aufiero et al., 2019), to predict the circRNAs that have translation capacity. Interestingly, circRNA is well known for its covalently closed RNA molecule without 5′caps and 3′tails (Meng et al., 2017), therefore, circRNA must be translated in cap-independent translation initiation mechanisms. Two mechanisms of circRNA translation have been reported: internal ribosome entry site (IRES)-dependent initiation of translation and m6A-dependent initiation of translation. The m6A-driven translation of circRNAs was widespread. Yang et al. (2017) predicted that hundreds of circRNAs can potentially be translated into proteins by the analysis of polysome profiling, computational prediction, and mass spectrometry. The m6A-driven translation of circRNA may require initiation factor eIF4G2 and m6A reader YTHDF3, and can be enhanced by methyltransferase METTL3/14, inhibited by demethylase FTO (Yang et al., 2017). Yang et al. (2017) inserted a short fragment (19 nt) containing different copies of consensus m6A motifs (RRACH) before the start codon of circRNA reporter in 293 cells, and the protein was then translated and detected.

It only requires one single m6A site to have the same translation efficiency compared to circRNA with two m6A sites (Yang et al., 2017). In human papillomavirus (HPV), m6A-modified circE7 is translated to E7 oncoprotein (Zhao et al., 2019).

Further analysis found that m6A regulated its translation through recognition by YTHDF3 and eIF4G2 (Di Timoteo et al., 2020). This study suggested that the two cap-independent translations of circRNA might interact. However, the relationships between the two cap-independent translations need further investigation. Interestingly, it has been reported that m6A-mediated circRNA translation increases under stress, e.g., heat shock conditions. The underlying mechanism is presumably due to the translocation of YTHDF2 from the cytosol into the nucleus, subsequently blocking the m6A “eraser” FTO (Yang et al., 2017; Zhou et al., 2015). The m6A-mediated circRNA translation may serve as a host-defense mechanism in cellular stress response.

A summary of the m6A impacts on circRNA biogenesis and regulation is illustrated in Scheme 2.

## Association between m6A modification and circRNAs in the inflammatory responses

Emerging evidence suggests that m6A modifications on the host gene or the mature circRNAs may play essential roles in inflammatory responses and innate immunity against noxious stimuli.

M6A-modified natural circRNAs are abundant based on the studies of m6A-methylated RNA immunoprecipitation sequencing (MeRIPseq) and m6A-circRNA microarray data (Roundtree et al., 2017; Zhao et al., 2022).

### M6A modification of circRNAsplays a crucial role in regulating inflammation and innate immunity and potentially impacts various diseases

1) M6A as a mark to differentiate endogenous vs. exogenous circRNAs.

Endogenous circRNAs form 16–26 bp imperfect RNA duplexes to resist the double-stranded RNA (dsRNA)-activated protein kinase (PKR) in innate immunity (Liu et al., 2019). Mammalian cells distinguish between foreign and endogenous circRNAs based on their m6A modifications. For example, different levels of m6A modification were detected in circRNA generated by ZKSCAN1 introns (circSELF, referring to endogenously generated circRNA), but not autocatalytic splicing (circFOREIGN, referring to the circRNAs to be removed by autocatalysis). M6A modification marked circRNA as “SELF”. Another type of foreign circRNAs, such as the viral RNAs, may evoke the antiviral response. Endogenous circRNAs with the m6A modifications can be recognized by m6A readers (e.g., YTH proteins) as the “self” molecules, subsequently escape from the immune surveillance and reduce interferon production (Chen YG. et al., 2019). For example, circSELF can evade innate immunological surveillance via YTHDF2-mediated suppression. YTHDF2 binds m6A-modified circRNAs, preventing their detection by dsRNA sensor RIG-I and the subsequent stimulation of innate immunity (Chen YG. et al., 2019). Unmodified circRNA activates RIG-I in the presence of K63-polyubiquitin to cause MAVS filamentation, IRF3 dimerization, and interferon production.

Not all the circRNAs have m6A modifications. However, more than 1,000 endogenous circRNAs have been reported to possess m6A modifications. The m6A-modified circRNAs are cell-type specific and often lie on exons that are not m6A-modified in their corresponding mRNAs (Zhou et al., 2017).

As mentioned above, certain exogenous circRNAs have been reported to induce innate immunity genes and confer protection against viral infection (Chen et al., 2017). On the other hand, m6A modification facilitates circRNA translation and helps foreign circRNAs escape immune surveillance, as m6A is a “self” mark in these circRNAs. As little as 1% m6A modifications in artificial circRNA can reduce the induction of innate immunogenicity. 100% m6A modifications in artificial circRNA completely abrogated the induction of innate immunogenicity.

2) M6A modification on circRNAs plays a role in viral infection-induced immunity.

M6A expression on endogenous RNAs has been demonstrated to play a role after viral infection (Winkler et al., 2019), suggesting that m6A regulates innate immunity

Consistently, recent reports demonstrate that circRNA encoding antigenic protein sequences delivered by a charge-altering releasable transporter can effectively serve as both an adjuvant and an immunogen, inducing potent cellular immunity and serving as a therapeutic vaccine. One key factor in generating circRNA vaccine is to avoid m6A modification. For example, mice intranasally immunized with either m6A-modified or -unmodified circOVA, the lungs were analyzed for antigen-specific T cell responses. M6A modification has been shown to promote the translation of circRNAs (Yang et al., 2017); however, m6A abrogates circRNA immunity (Chen YG. et al., 2019). Naked delivery of m6A-modified circOVA did not induce any OVA-specific T cell responses (Chen YG. et al., 2019). The unmodified circOVA group generated potent OVA-specific CD8 T cell responses (Chen YG. et al., 2019).

3) M6A modification on circRNAs plays a role in bacterial infection-induced immunity.

Fewer studies have been performed on bacterial infections. The first question to answer is whether bacterial infection modifies m6A modifications of circRNAs. Yu (2024) discovered a new circHIF1α, whose secretion into exosomes was significantly decreased after bacterial infections (Yu et al., 2024). Additionally, exosomal circHIF1α reduces bacterial infection both *in vitro* and *in vivo* and suppresses the growth of receptor cells (Yu et al., 2024). CircHIF1α interacted with the KH domain of IGF2BP3 in an m6A-modified manner, which arrests the cells at the G1/S phase through the interaction between the regulator of Chromosome Condensation 2 (RCC2) and γ-H2AX protein (Yu et al., 2024). M6A-modified exosome-derived circHIF1α mediates DNA damage and arrests G1/S transition phase to resist bacterial infection in bacteremia, therefore, exosomal circHIF1α potentially serves a unique therapeutic target for bacterial infection (Yu et al., 2024).

Lipopolysaccharide (LPS), an endotoxin produced by Gram-negative bacteria, may affect the host’s m6A modification under oxidative stress. Previous report shows that YTHDF2 can mitigate LPS-induced inflammation in mouse macrophages via reducing MAP2K4 and MAP4K4 mRNA levels and inhibiting the MAPK and NF-κB pathways. In addition to LPS, the exotoxin of Gram-positive bacteria can also regulate intestinal m6A levels. The *Clostridium perfringens* beta2 (CPB2) toxin induces a notable surge in overall m6A RNA methylation levels in porcine intestinal epithelial (IPEC-J2) cells. This m6A modification may be associated with CPB2-triggered inflammatory and antiviral responses, potentially via the Wnt signaling pathway (Zhang et al., 2021; Yang et al., 2021). CPB2 elevated m6A and METTL3 levels in IPEC-J2 cells via enhancing the TLR2/NF-κB pathway, exacerbating CPB2-induced inflammatory responses in these cells (Zhang et al., 2022). M6A may serve as a conductor in the orchestration of host-microbiome interactions, working in synergy with circRNAs, chromatin remodeling, and histone modifications (Zhang et al., 2022; Zhuo et al., 2022).

## Conclusion

Our understanding of how m6A modification regulates circRNA, particularly in the field of inflammation and immunity, remains a rapidly growing area. M6A potentially adds additional regulation on the biological function of circRNAs in the development of inflammatory responses and innate immunity against sterile or infectious stimuli. M6A-modified circRNAs may serve as novel diagnostic and therapeutic targets in various human diseases, including inflammatory processes. There is still a long way to go to understand m6A’s regulatory mechanisms and subsequent biological functions in circRNA research.
